# Study of correlation between forced vital capacity and demand for healthcare services in severe asthmatics

**DOI:** 10.1186/s40248-015-0020-5

**Published:** 2015-07-22

**Authors:** J.F. Castelluccio, R. Stirbulov, E.A. Perez, J.C.M. Oliveira, C.F. Donner, L.V.F. Oliveira, Z. Rasslan

**Affiliations:** Assistant Professor Internal Medicine Department, Santa Casa School of Medicine and Hospitals of São Paulo, São Paulo (SP), Brazil; Associate Professor Internal Medicine Department – Pneumology, Santa Casa School of Medicine and Hospitals of São Paulo, São Paulo (SP), Brazil; Master Degree Program in Research in Surgery of Santa Casa School of Medicine of Sao Paulo (FCMSCSP), Sao Paulo (SP), Brazil; Rehabilitation Sciences PhD Program, Nove de Julho University, Sao Paulo, Brazil; Mondo Medico, Multidisciplinary and Rehabilitation Outpatient Clinic, Borgomanero, NO Italy; Rehabilitation Sciences Master and PhD Program, Nove de Julho University, Sao Paulo, Brazil; Assistant Professor Internal Medicine Department, Santa Casa School of Medicine and Hospitals of São Paulo, São Paulo (SP), Brazil; Rua Itapicuru, 380 apto 111 Perdizes, CEP 05006-000 Sao Paulo, SP Brazil

**Keywords:** Asthma, Healthcare utilization, Vital capacity

## Abstract

**Background:**

Involvement of the small airways may be related to increased severity and increased demand for health care services and incurring in high costs, private or for the healthcare system. The hyperinflation consequent to this involvement reduces lung volumes, such as FVC, FEV_1_ and SVC. The aim of this study was to evaluate the correlation between the predicted values of FVC, FEV_1_ and SVC with the demand for healthcare services by severe asthmatics.

**Methods:**

We retrospectively evaluated in order of arrival, the medical records of 98 patients with severe asthma, in step 4 treatment in the intercritical period of the disease, correlating the number of times each patient sought health care services represented by admissions to the ER, ICU and hospital wards due to asthma, in the year before the last spirometry and the predicted values of FVC, FEV_1_ and SVC.

**Results:**

Our sample showed a clear and significant negative correlation between the predicted values of FVC, FEV_1_ and SVC and demand for healthcare services.

**Conclusion:**

For this sample we conclude, that reduced forced vital capacity correlated with asthma severity, defined by greater demand for care in the ER, ICU and hospital ward and was more evident in women.

## Background

It is estimated that 10 % of asthma patients have the severe form of the disease with more frequent exacerbations and increased morbidity, resulting in increased demand for healthcare services such as admission to the emergency rooms (ER), to hospital wards and to intensive care units (ICU) incurring higher costs. Such direct and indirect costs are assigned to hospitalizations, drugs, school and work absenteeism [1]. In developing countries expenditures with severe asthma seriously affect household income contributing to the impoverishment of these patients [2].

The small airways have been considered as the primary site of obstruction to airflow in chronic inflammatory lung diseases such as severe asthma and differently from what was believed, these airways undergo the same inflammatory processes as the larger caliber ones. This involvement should be suspected whenever there are persistent symptoms such as chest tightness and cough, associated with absence of lung function impairment [3].

The assessment and early identification of patients prone to fatal or near-fatal events are paramount in management, prophylaxis, therapy and cost reduction in severe asthma. Spirometry is the most often used functional assessment to address and monitor obstructive pulmonary diseases such as asthma since the procedure is easy and noninvasive. Further, it correlates with obstruction of the small airways, has high reproducibility, low variability and lesser cost when compared to others. The Forced Expiratory Volume at first second (FEV_1_) %, Forced Vital Capacity (FVC)% and Slow Vital Capacity (SVC)% assess the function of the small airways and may be used as markers correlated with use of health care services [3].

### Objective

To evaluate the correlation between decreased FVC, FEV_1_ and SVC and demand for healthcare services, such as admission to ER, to hospital ward and to ICU in severe asthma.

## Methods

A retrospective evaluation was made of the last spirometric data of 114 patients out of a total of 740 asthmatic subjects. These were severe asthma patients enrolled at the outpatient clinic of Santa Casa de Sao Paulo, classified in order of arrival from 2005 to 2008. This study was submitted for review and approval by the Ethics and Human Research Committee, Santa Casa de Sao Paulo. The percentage of predicted FVC% and SVC% were the spirometry parameters used as a reference, in accordance with international guidelines for the execution of lung function tests established by the European Respiratory Society and American Thoracic Society [4] and Brazilian Society of Pneumology (SBPT) [5].

The spirometry measurements used corresponded to those of patients who were not defined as exacerbation patients. Exacerbation patients were those under regular treatment with inhaled corticosteroids and who required oral medication or those who were already using oral corticosteroids but who needed temporarily increased doses due to acute deterioration of their control condition [6]. We considered the number of times each patient sought healthcare services, such as admission to the ER, to hospital wards and ICU, because of asthma in the previous year and of the assessed spirometry parameters.

Correlation was established between the predicted values of FVC% and SVC% with use of the mentioned healthcare services. Other spirometry parameters such as predicted values of FEV_1_%, FEV_1_/FVC% and SVC-FVC difference were also evaluated. According American Thoracic Society (ATS), as inclusion criteria we adopted severe asthmatics [7] with spirometry performed in the intercritical period of the disease (out of exacerbation) and under full use of medication, compatible with the step 4 approved treatment by The Global Initiative for Asthma (GINA) [8] and guidelines of SBPT [9] (long lasting inhaled beta 2 adrenergic + long lasting inhaled corticosteroid). Exclusion criteria were other obstructive lung diseases or associated muscle, hematologic and cardiac diseases, asthma attack, current or prior smoking and obesity.

Spirometry was performed at the Lung Function Laboratory of the Santa Casa de Sao Paulo, by the same team of technicians. The device used was of the Koko brand, manufactured in 1998 by PDS Instrumentation Inc. Louisville, CO, USA, with a pneumotachograph, serial number 92528, coupled to a computer. We assessed the following spirometry parameters: 1. Percentage of predicted values of FVC%; 2. Percentage of predicted values of the SVC%; 3. Percentage of predicted values of FEV_1_%; 4. Percentage of predicted values of the ratio FEV/FVC; 5. % Difference SVC—FVC. We used SPSS (Statistical Package for Social Sciences), version 17.0, to obtain the results of statistical assessments. The study of the relationship between categorical variables was obtained by applying the Spearman and the Mann–Whitney correlation analyses for the entire sample. The adopted level of significance for tests and statistical analyses was 5 %.

## Results

We studied 114 patients, 16 out of these were excluded for concomitant Chronic Obstructive Pulmonary disease (COPD) (15 patients) and Interstitial lung disease (1 patient) resulting in 98 asthmatic patients (n = 98), followed for over two years, with a mean age of 42 ± 19.3 years, 45 men and 53 women. They were at stage 4 of treatment using long lasting corticosteroids inhalers (budesonide-600 μg/day) and beta 2 adrenergic (formoterol-24 μg/day) or equivalent doses of other drugs of the same class. There was no statistical difference regarding age in both genders and spirometric variables, indicating homogeneity of the sample (Table 1).

**Table 1:** Means of ages and spirometry variables per gender of the studied population of 98 severe asthmatics patients

	Men (n = 45)	Women (n = 53)	Total (n = 98)	
Variables	Mean ± SD	Mean ± SD	Mean ± SD	*p*
Age	42.6 ± 21.1	41.4 ± 17.7	42.0 ± 19.3	0.69
%FVC	80.3 ± 23.4	75.0 ± 19.2	77.4 ± 21.3	0.43
%SVC	78.5 ± 19.8	75.1 ± 17.7	76.7 ± 18.7	0.46
%FEV_1_	55.7 ± 21.3	54.0 ± 21.0	54.8 ± 21.0	0.81
%FEV_1_/FVC	67.4 ± 17.0	69.0 ± 15.4	68.3 ± 16.1	0.73
%SVC/FVC	1.80 ± 8.15	0.11 ± 8.16	0.77 ± 8.17	0.59

Source: Pulmonary Function Laboratory, Santa Casa de Sao Paulo.

Means and Standard Deviation of ages and spirometry variables % Predicted values: FVC, Forced Vital Capacity; SVC, Slow vital Capacity; FEV_1_ Forced Expiratory Volume at 1st second; n (men) = 45, n (women) = 53, n (total) = 98 Mann–Whitney Test SD, Standard Deviation

Correlations between demand for ER and % of predicted values of FVC, SVC, FEV_1_, although weak, were significant (p < 0.05), the same also for the ratio demand for ER + ICU + HWR as a whole. (Table 2)

**Table 2:** Correlation between spirometry values and demand for healthcare services of the studied population of 98 severe asthmatics patients

Variable	%FVC		%SVC		%FEV_1_		%FEV_1_/FVC		%SVC-FVC	
	r	p	r	p	r	p	r	p	r	p
ER/year	−0.25	0.01	−0.22	0.02	−0.23	0.01	−0.15	NS	−0.06	NS
ICU	−0.15	NS	−0.18	NS	−0.16	NS	−0.07	NS	−0.03	NS
HWR	0.15	NS	0.13	NS	0.04	NS	0.15	NS	0.03	NS
ER + ICU + HWR	−0.25	0.01	−0.21	0.03	−0.23	0.01	−0.12	NS	−0.08	NS

% - Predicted values: FVC, Forced Vital Capacity; SVC, Slow Vital Capacity; FEV_1_ Forced Expiratory Volume at first second; NS,- not significant; ER/year, demand for emergency room /year; ICU, demand for Intensive Care Unit; HWR, referral to hospital wards; ER+ ICU+ HWR, referral to emergency room + Intensive care unit + Hospital wards. n = 98 (53 women and 45 men)

Sample stratification in genders showed negative and significant correlations in women between demand for ER and values of spirometry (FVC%, %SVC, FEV_1_% and FEV_1_/FVC% with r =− 0.44, p = 0.001, r = − 0.41, p = 0.002, r = − 0.50, p <0.001, r = − 0.42, p = 0.002, respectively), as well as the need for ICU (%FVC, %SVC, with %FEV_1_ r = − 0.38, p = 0.004, r = − 0.36, p = 0.008, r = − 0.27, p = 0.048 respectively), hospital ward (%FVC, %FEV_1_ and r = − 0.31, p = 0.022, r = − 0.34, p = 0.012 respectively).

There are also significant and negative correlations when taking into account the demand for all healthcare services (ER + ICU + HWR) with (%FVC, %SVC, %FEV_1_ and %FEV_1_/FVC with r = − 0.43, p = 0.001, r = − 0.38, p = 0.005, r = − 0.49, p <0.001, r = − 0.38, p = 0.004, respectively) (Table 3). No significance was found between ER, the need for ICU, hospital ward and ICU and all three together ER + ICU + HWR and the values of spirometry % (SVC-FVC) (r = − 0.12, r = − 0.11, r = − 0.25, r = − 0.21), respectively. A similar situation occurred with the demand for ICU and hospital ward in relation to the variable %FEV_1_/FVC (r = − 0.03, r = − 0.25), respectively (Table 3). In the male gender no significant values for any of the spirometry variables studied and demand for healthcare services were observed. (Table 3).

**Table 3:** Correlation between spirometry values and demand for healthcare services by gender of the studied population of 98 severe asthmatics patients

Variables	Gender	% FVC		% SVC		% FEV_1_		% FEV_1_/FVC		% SVC-FVC	
		r	p	r	p	r	p	r	p	r	p
ER	M	−0.03	NS	−0.02	NS	−0.11	NS	−0.18	NS	−0.09	NS
	W	−0.44	0.001	−0.41	0.002	−0.50	0.001	−0.42	0.002	−0.12	NS
ICU	M	− 0.11	NS	− 0.02	NS	−0.008	NS	−0.11	NS	−0.14	NS
	W	−0.38	0.004	−0.36	0.008	−0.27	0.048	−0.03	NS	−0.11	NS
HWR	M	−0.01	NS	−0.008	NS	−0.02	NS	−0.13	NS	−0.01	NS
	W	−0.31	0.022	−0.25	NS	−0.34	0.012	−0.25	NS	−0.25	NS
Er + ICU + HWR	M	−0.03	NS	−0.03	NS	−0.09	NS	−0.19	NS	−0.07	NS
	W	−0.43	0.001	−0.38	0.005	−0.49 < 0.001		−0.38	0.004	−0.21	NS

M, Men; W, Women; ER, demand for the emergency room / year; ICU, demand for admission to the Intensive Care Unit; HWR, Hospital ward / year; FVC, forced vital capacity; SVC, slow vital capacity; FEV_1_,forced expiratory volume in 1st second; NS, not significant

## Discussion

Severe or refractory asthma affects a small percentage of asthmatics (probably less than 10 %), nevertheless it represents a challenging condition for the patient and the physician in charge [10]. Asthma is physiologically characterized by flow limitation, bronchial hyperreactivity, airway narrowing and/or occlusion, also by loss of lung elasticity and air trapping each of which may be assessed and monitored by spirometry.

Severe asthma correlates with an increased number and greater severity of exacerbations, therefore a frequent demand for healthcare services encompassing admission at the ER, hospital wards and need for ICU contributing to increased mortality, and private and health system expenditures. Bahadori *et al.* found in their studies that the average annual direct cost of patients with severe asthma in the United States was 1.3 times higher than that of those with moderate asthma and 1.7 times that of mild asthma. In Spain, between 1994 and 1995, the cost of a patient with severe asthma was three times higher than that of one with moderate asthma and 5 times that of one with mild asthma [1].

Early assessment and identification of patients with severe asthma, who therefore are prone to fatal or near-fatal events is paramount in management, prophylaxis, therapy, and cost reduction.

Our study showed that there is negative and significant correlation (p <0.05) between the demand for health care services and the predicted values of FEV_1_, FVC and SVC in severe asthmatics (Table 2). These results are consistent with data from literature which also report a decrease in FEV_1_ associated with an increased risk of asthma exacerbations, as well as an inverse relation between its value and higher risk of fatal or near-fatal asthma [11]. The TENOR study, which established a risk score that accurately predicts the demand for healthcare services by adults with severe asthma found that FEV_1_ and FEV_1_/FVC ratio were not as predictive as the FVC. It detected that the reduction in FVC may be an important variable, since in addition to showing air trapping in the obstructive disease, it may also disclose restriction associated with obesity. Furthermore, air trapping and lung hyperinflation are associated with a greater frequency of severe asthma exacerbations [11].

Overall, 239 (8.5 %) of the TENOR subjects studied reported a hospitalisation or an ED visit for asthma at 6-month follow up. The number of points assigned is positively associated with the percentage of patients with a hospitalization or an ED visit in that component category.

Despite the fact that the TENOR risk score has limitations related to external validity, or generalisability, because the study population does not represent all asthma patients but only severe or difficult-to-treat adult asthma patients, the authors concluded that the identification of clinical factors in patients with severe or difficult-to-treat asthma that are associated with future healthcare use may improve asthma patient management and ultimately reduce the burden of disease on the overall healthcare system [11].

Sorkness *et al.* [12] also showed that air trapping is prevalent in severe asthma as shown by reduced FVC over the entire interval FEV_1_/FVC. Despite these associations, patients with fatal or near-fatal asthma may show no changes in the baseline spirometry test [13]. This may explain the weak correlation in the ratio, %FEV_1_/FVC in relation to demand for healthcare services (need for ICU and hospital ward) respectively, as found herein (Table 2).

The results of this study (see Table 2) showed the correlations between the values of spirometry (FVC%, FEV_1_% and SVC%) and asthma severity criteria represented by the demand for admission in the ER, need for ICU or hospital ward as well as the total showed weak, negative correlation when both genders were evaluated together. Even though weak, this correlation showed significance for the demand for care in the ER and the sum of admissions (ICU+ ER + HWR). These findings reinforce the idea that reductions in these variables may indicate asthma severity and agree with data of other authors showing that the best predictors of demand for healthcare in the ER by patients with severe asthma were the reduction of FEV_1_% and FVC% [11, 12, 14, 15].

The analysis in relation to gender revealed no significance for % FVC predicted values, %SVC, %FEV_1_, %FEV_1_/FVC and % (SVC-CVF) and the demand for health care services (ER admission, need for ICU referral to hospital ward, individually or together (ER + ICU+ HWR) due to asthma in males (Table 3). These findings may be explained by the existence among participant men of a subset of “bad perceivers” who although at spirometry present values compatible with fixed airway obstruction, do not perceive symptoms and therefore do not seek medical care [16].

Findings by Teeter and Bleecker [17] demonstrated that 17 % of the patients studied did not show any symptoms, despite their low spirometry values.

The female gender, included in our study, showed a negative correlation, more consistent and significant (p <0.05) between the spirometry values %FVC, %SVC, %FEV_1_, %FEV_1_/FVC and %FVC- SVC and demand for healthcare services: admission to ER and (ER + ICU+ HWR) as a whole (Table 3). The findings of this study are similar to those of other authors, which showed that the female gender more often seek healthcare services [18]. Prescott *et al.* [19] found that women had a 70 % higher risk of being admitted to the hospital due to asthma when compared to men. Another study showed that asthmatic women were hospitalized 2.5 to 3 times more often than men [20].

The correlations between values of %FVC, %SVC, %FEV_1_ and %FEV_1_/FVC and demand for healthcare services in asthmatic women (ER + ICU + HWR) were stronger and significant (r = − 0.43 p = 0.001) (r = − 0.38, p = 0.005), (r = − 0.49, p = <0.001) and (r = − 0.38, p = 0.004) compared to men (Table 3). These findings suggest that in women asthma is potentially more serious than in men.

Gelb *et al*. [21] showed that lung elasticity in patients with chronic persistent asthma contributes significantly to clinical complications, as is the case of near-fatal asthma. Loss of lung elasticity was associated with increasing age, disease duration and severity of expiratory airflow limitation, using as a signal the %FEV_1_ post-bronchodilator. However, this study shows, as a limitation, absence of aspects related to FVC behavior. This author further stresses that for many reasons, studies of pulmonary function are not obtained in most patients when they are clinically stable, before and after episodes of near-fatal asthma [22]. In our study, spirometry values were considered during the intercritical period of the disease and findings show that in women forced vital capacity, slow vital capacity and forced expiratory volume in the first second correlate negatively and significantly with the demand for healthcare services (Table 3).

More specifically, FVC and FEV_1_ were significant in relation to admissions to ER, need for ICU, hospital ward individually and/or together (ER + ICU + HWR), indicating its usefulness for the assessment of severe asthma in the intercritical period of the disease (off exacerbation) in daily practice (Table 3).

Airway occlusion is the most serious type of obstruction and assessing changes in FVC may supply information about the pathophysiology of asthma, which does not appear when considering changes in FEV_1_. We stressed FVC, which showed significant correlation with the use of healthcare services for women with severe asthma (Table 3). It was noted that in women, the correlation (r), although weak, showed higher and significant negative values (p <0.05) when compared to men, indicating that this is not due to chance.

Scientific evidences have shown several phenotypes of severe asthma, requiring the integration of measurements of airflow limitation, airway reactivity, small airway disease, lung elasticity and perception of dyspnea to explain and diagnose properly this heterogeneous disease [20, 23].

## Conclusions

We conclude that in severe asthmatics patients reduced FVC correlated with asthma severity, defined by greater demand for care in the ER, ICU and hospital ward, and was more evident in women.

## References

[1] Bahadori K, Doyle-Waters MM, Marra C, Lynd L, Alasaly K, Swiston J (2009). Economic burden of asthma: a systematic review. BMC Pulmonary Medicine.

[2] Franco R, Nascimento HF, Cruz AA, Santos AC, Souza-Machado C, Ponte EV (2009). The economic impact of severe asthma to low-income families. Allergy.

[3] Cohen J, Postma DS, Vink-Klooster K, van der Bij W, Verschuuren E, Ten Hacken NH (2007). FVC to slow inspiratory vital capacity ratio: a potential marker for small airways obstruction. Chest.

[4] Miller MR, Hankinson J, Brusasco V, Burgos F, Casaburi R, Coates A (2005). SERIES “ATS/ERS Task Force: Standardisation of Lung Function Testing”. Eur Respir J.

[5] Pereira CAC (2002). II Consenso Brasileiro de Espirometria. J Pneumol.

[6] Abraham B, Anto JM, Barreiro E, Bel EHD, Bonsignore G, Bousquet J (2003). The ENFUMOSA cross-sectional European multicentre study of the clinical phenotype of chronic severe asthma. Eur Respir J.

[7] Society AT (2000). Proceedings of the ATS workshop on refractory asthma current understanding, recommendations, and unanswered questions. Am J Respir Crit Care Med.

[8] Bateman ED, Hurd SS, Barnes PJ, Bousquet J, Drazen JM, FitzGerald M (2008). Global strategy for asthma management and prevention: GINA executive summary. Eur Respir J.

[9] Sociedade Brasileira de Pneumologia e Tisiologia (2006). IV Diretrizes Brasileiras para o Manejo da Asma. J Bras Pneumol.

[10] Wenzel S (2006). Physiologic and pathologic abnormalities in severe asthma. Clin Chest Med.

[11] Miller MK, Lee JH, Blanc PD, Pasta DJ, Gujrathi S, Barron H (2006). TENOR risk score predicts healthcare in adults with severe or difficult-to-treat asthma. Eur Respir J.

[12] Sorkness RL, Bleecker ER, Busse WW, Calhoun WJ, Castro M, Chung KF, Sorkness RL, Bleecker ER, Busse WW, Calhoun WJ, Castro M, Chung KF (2008). Lung function in adults with stable but severe asthma: air trapping and incomplete reversal of obstruction with bronchodilation. J Appl Physiol.

[13] Madison JM, Irwin RS. Identifying patients at risk for fatal asthma. Uptodate 19.3. [on line]. [Access 2011 Dec 14]. Available from: http://www.uptodate.com/contents/identifying-patients-at-risk-for-fatal-asthma

[14] Gelb AF, Zamel N (1973). Simplified diagnosis of small-airway obstruction. N Engl J Med..

[15] Osborne ML, Pedula KL, O’Hollaren M, Ettinger KM, Stibolt T, Buist AS (2007). Assessing future need for acute care in adult asthmatics: the Profile of Asthma Risk Study: a prospective health maintenance organization-based study. Chest.

[16] Souza-Machado A, Cavalcanti MN, Cruz AA (2001). Má percepção da limitação aos fluxos aéreos em pacientes com asma moderada a grave. J Pneumol..

[17] Teeter JG, Bleecker ER (1998). Relationship between airway obstruction and respiratory symptoms in adult asthmatics. Chest.

[18] Chhabra SK, Chhabra P (2011). Gender differences in perception of dispnea, assessment of control, and quality of life in asthma. Journal of Asthma.

[19] Prescott E, Lange P, Vestbo J (1997). Effect of gender on hospital admissions for asthma and prevalence of self-reported asthma: a prospective study based on a sample of the general population. Copenhagen City Heart Study Group. Thorax..

[20] Skobeloff EM, Spivey WH, Clair SS, Schoffstall JM (1992). The influence of age and sex on asthma admissions. JAMA.

[21] Gelb AF, Schein A, Nussbaum E, Shinar CM, Aelony Y, Aharonian H (2004). Risk factors for near-fatal asthma. Chest.

[22] Lee YM, Park JS, Hwang JH, Park SW, Uh ST, Kim YH (2004). High-resolution CT findings in patients with near-fatal asthma: comparison of patients with mild-to-severe asthma and normal control subjects and changes in airway abnormalities following steroid treatment. Chest..

[23] Wenzel SE, Schwartz LB, Langmack EL, Halliday JL, Trudeau JB, Gibbs RL (1999). Evidence that severe asthma can be divided pathologically into two inflammatory subtypes with distinct physiologic and clinical characteristics. Am J Respir Crit Care Med..

